# Purification of Bioactive Natural Antibodies to PD-1 Known as Regulatory Rheumatoid Factor from Human Blood Plasma

**DOI:** 10.3390/antib15050098

**Published:** 2026-10-19

**Authors:** Daria Menshikova, Alexey Terentiev, Pavel Ivanov, Alexandr Cherednichenko, Liubov Beduleva

**Affiliations:** 1 Laboratory of Molecular and Cellular Immunology, Department of Immunology and Cell Biology, Udmurt State University, 1 Universitetskaya St, Izhevsk 426034, Russia; alts4m@yandex.ru (A.T.); aka.ven1k@gmail.com (P.I.); lvbeduleva@gmail.com (L.B.); 2 Department of Physical and Colloid Chemistry, Peoples’ Friendship University of Russia Named After Patrice Lumumba (RUDN University), 6 Miklukho-Maklaya St, Moscow 117198, Russia; cherednichenko-ag@pfur.ru; 3 Laboratory of Biocompatible Materials, Udmurt Federal Research Center UB RAS, 34 T. Baramzinoy St, Izhevsk 426067, Russia

**Keywords:** regulatory rheumatoid factor, natural antibodies to PD-1, purification

## Abstract

Background/Objectives: Regulatory rheumatoid factor (regRF) is a recently discovered population of natural anti-idiotypic antibodies carrying a shared paratope specific to the PD-1 molecule. Normally, regRF exerts a cytotoxic effect on PD-1-expressing T lymphocytes, including autoreactive ones, thereby controlling their expansion. The specificity for PD-1 indentifies regRF as a promising therapeutic agent for suppressing autoimmune reactions via the PD-1 pathway. The aim of this study was to develop gentle methods for regRF isolation from healthy blood plasma, which would preserve the bioactivity of regRF. Methods: Ammonium sulfate precipitation was used for crude regRF purification. A search for suitable affinity sorbents was carried out. The purified regRF was tested for its bioactivity. Results: Ammonium sulfate precipitation at 35% saturation, followed by extraction with 28% saturated ammonium sulfate solution, allowed the separation of regRF from a portion of non-target IgG and high-molecular-weight fibrinogen. RegRF showed high affinity for Cibacron Blue and no binding to Protein A. Sequential affinity chromatography on immobilized Cibacron Blue and Protein A enabled the purification of regRF from non-target immunoglobulins and low-molecular-weight fibrinogen. The purified regRF samples showed binding to PD-1 and reduced the PD-1+CD4+ T lymphocyte count in vitro. Conclusions: Thus, we have identified sorbents and conditions for the purification of biologically active regRF.

## 1. Introduction

We have previously discovered the new autoimmunity downregulation factor called regulatory rheumatoid factor (regRF) [1,2,3]. Studies of regRF specificity have shown that these antibodies possess an individual paratope and a shared paratope [1]. Individual paratopes of regRF molecules are specific to the unique idiotypes of antibodies and T-cell antigen receptors, allowing for idiotype-anti-idiotypic interactions between regRF and lymphocytes [1]. The shared paratope of regRF is specific to the PD-1 molecule [4]. As a dual-reactive antibody, regRF ensures cross-linking between PD-1 and T-cell antigen receptors, which is essential for PD-1-mediated signaling [5]. RegRF exerts cytotoxic action on PD-1 expressing CD4+ T lymphocytes [4], limiting the expansion of autoreactive T lymphocytes and promoting the maintenance of natural tolerance.

The cytotoxic effect of regRF on PD-1-expressing T lymphocytes suggests that regRF can be used to suppress autoimmune reactions via the PD-1 pathway. Stimulation of regRF production reduced the symptoms of disease and decreased tissue damage in experimental autoimmune arthritis [3], thyroiditis [6], encephalomyelitis [7], and gastritis models [8].

Nevertheless, upregulated regRF production might be ineffective for patients with defects in regRF structure. Replacement therapy with donor-derived regRF could be a more appropriate approach for such patients. RegRF may also prove effective in correcting adverse autoimmune reactions in cancer patients after targeted therapy with blocking monoclonal antibodies against PD-1/PD-1L.

The advancement of regRF toward clinical application requires the development of methods for purifying regRF from blood plasma. Limited knowledge of the physicochemical properties of regRF poses challenges in selecting appropriate purification methods. Furthermore, it is essential that the isolation process preserves the biological activity of regRF. The current study aimed to develop methods for purifying bioactive regRF from human blood plasma.

## 2. Materials and Methods

### 2.1. Blood Components

Quarantined healthy human blood plasma was provided by the Republic Blood Transfusion Station (Izhevsk, Russia). Lymphocytes were obtained from the peripheral blood of 9 healthy volunteers collected at clinical laboratories. Volunteers provided informed consent for the research use of their blood samples.

### 2.2. Institutional Review Board Statement

The study was conducted in accordance with the Declaration of Helsinki, and the protocol was approved by the Bioethics Committee of Udmurt State University (Project identification code 2502) on 20 January 2025.

### 2.3. Precipitation of the IgG Fraction from Blood Plasma with 35%-Saturated Ammonium Sulfate Solution

Human blood plasma was centrifugated at 2300 *g* for 15 min. The supernatant was transferred to a beaker, and ammonium sulfate was slowly added to achieve 35% saturation. After complete dissolution of the salt, the mixture was incubated for 1 h and then centrifuged at 2300 *g* for 15 min. The supernatant was discarded, and the pellet was washed twice with a 35%-saturated ammonium sulfate solution. The final pellet represented the IgG fraction of human blood plasma.

### 2.4. Extraction of RegRF from the IgG Fraction of Human Blood Plasma

The extraction of regRF from the IgG fraction obtained by precipitation of blood plasma with 35% saturated ammonium sulfate was performed using two approaches: parallel and sequential (Figure S1).

For the parallel extraction, the IgG precipitate was divided into three equal portions, and 31.5%, 29.7%, or 28% saturated ammonium sulfate solution was added to these portions (Figure S1A). The suspensions were stirred for 10 min and then centrifuged at 2300 *g* for 15 min. The pellets and supernatants were collected and buffer-exchanged into 0.15 M PBS, pH 7.3 using ultrafiltration. The resulting solutions were tested for regRF.

In the serial extraction, the pellet was first resuspended in a 31.5% saturated AS solution. The supernatant was collected and kept for analysis. The pellet, which did not dissolve at 31.5% saturation, was treated with an ammonium sulfate solution at 29.7% saturation. The supernatant was also collected and kept for analysis. Lastly, the remaining pellet was resuspended in a 28% saturated AS solution, which resulted in a pellet and a supernatant (Figure S1B). Both were analyzed.

### 2.5. Measurement of RegRF

The regRF titer was determined in an agglutination test using human IgG-loaded tanned human erythrocytes. For this, group O human erythrocytes, provided by the Republic Blood Transfusion Station, were fixed with 1% glutaric dialdehyde. The erythrocytes were thoroughly washed. Then a 10% erythrocyte suspension was incubated with an equal volume of a 0.008% tannin solution in PBS for 10 min at RT. After washing, the tanned erythrocytes were loaded with IgG. For sensitization, a 1.5% suspension of tanned erythrocytes in PBS (pH 6.4), containing 0.1 mg/mL human IgG, was incubated for 20 min at RT. The erythrocytes were washed with PBS (pH 7.3) containing 0.2% BSA. Twofold serial dilutions of serum were prepared and put into wells in aliquots of 50 μL. The same amount of human IgG-loaded 1.5% erythrocyte suspension was then added. Agglutination results were read after 3 h.

### 2.6. RegRF Depletion

Depletion of regRF was performed on a dense pellet of tanned IgG-loaded erythrocytes for 70 min at RT.

### 2.7. Cibacron Blue Chromatography

A column with dimensions of 16 × 200 mm was packed with 15 mL Cibacron Blue-Agarose sorbent (BlueBeads 6FF) (Smart-Lifesciences, Changzhou, China). The column was equilibrated with 0.02 M phosphate buffer (pH 7.4). A volume of 3 mL of the regRF-containing IgG fraction or 3 mL of defibrinated blood plasma was loaded. Elution was performed using a stepwise gradient of 2 M KCl in equilibration buffer. Several step-gradient elution schemes were evaluated, and the one providing optimal resolution and separation was selected. In the selected elution scheme, the mobile phase composition was varied in four discontinuous steps as follows: 0% 2 M KCl (0–40 mL), 5% 2 M KCl (40–60 mL), 20% 2 M KCl (60–80 mL), and 50% 2 M KCl (80–100 mL). Fractions were concentrated by ultrafiltration using membrane centrifugal concentrators (JetSpin Centrifugal Filter, Guangzhou Jet Bio-Filtration Co., Guangzhou, Ltd., Guangzhou, China) and kept for further procedures and analysis.

### 2.8. Protein A Chromatography

A protein A Sepfast HR column (Biotoolomics, Consett, UK), 5 mL, was equilibrated with 0.15 M PBS (pH 7.0). Then, 1 mL of the regRF-containing IgG fraction in equilibration buffer was loaded. The elution of bound species was performed by changing the buffer to 0.1 M glycine–HCl, pH 2.7. The eluted fractions were neutralized by adding 1 M Tris-HCl, pH 9.0. Buffer exchange into PBS was performed by ultrafiltration using membrane centrifugal concentrators (JetSpin Centrifugal Filter, Guangzhou Jet Bio-Filtration Co., Ltd., Guangzhou, China) and the samples were kept for further analysis.

### 2.9. Immunoblotting

Each protein sample was transferred from PAG to a nitrocellulose membrane (GE Healthcare, Amersham Bioscience, Amersham, UK) with a 0.45 µm pore diameter using a semi-dry transfer method, at 100 mA for 1 h in buffer containing 25 mM Tris-HCl and 0.2 M glycine. Before primary antibody incubation, the membranes were blocked with 5% (*w*/*v*) nonfat dried milk for 40 min and washed three times with PBS/Tween-20 (PBST). The membranes were then incubated with polyclonal rabbit antibodies specific for one of IgG subclasses (IgG1, IgG2, IgG3) (Cloud-Clone Corp., Wuhan, China) and monoclonal antibodies to IgG4 (Cloud-Clone Corp., Wuhan, China) in PBST for 30 min. After incubation, the membranes were washed three times with PBST and probed with secondary donkey anti-rabbit horseradish peroxidase-conjugated antibodies (BioLegend, San Diego, CA, USA) or anti-mouse peroxidase-conjugated antibodies (BioLegend, San Diego, CA, USA) for 30 min. Nonspecifically bound antibodies were removed by washing three times in PBST. Specific immunostaining was developed in DAB Substrate Solution (Termo Fisher Scientific, Waltham, MA, USA).

### 2.10. The Study of the Specificity of RegRF-Containing Fractions to PD-1 by ELISA

Plates (Corning-Costar, Acton, MA, USA) were coated overnight at 4 °C with 50 µL of human recombinant PD-1 (ACROBiosystems, Newark, DE, USA) (10 µg/mL in 0.15 M PBS. Plates were blocked with 200 µL of 0.15 mol/L PBS/1% BSA/Tween-20. Then, 100 µL of regRF-containing samples were added to the wells and incubated for 1 h at RT. The plates were then incubated for 1 h at RT with 100 µL of goat anti-human IgG conjugated to horseradish peroxidase (IMTEC, RF). Then, the substrate mixture (5 mL citrate buffer solution [pH 5.0]/3 mg ortho-phenylenediamine/15 µL 3% H_2_O_2_) was added. Absorbance was read after 15 min at 492 nm. All samples were analyzed in duplicate. The test sample ODA was calculated as the difference between the mean ODA of the test sample wells and the mean ODA of the negative-control wells. The negative control wells contained buffer instead of the test sample, while all other assay components were added as described above.

A CB2 IgG fraction devoid of regRF, obtained by fractionation of regRF-enriched IgG on a BlueBeads 6FF column, was used as a control for nonspecific IgG binding.

### 2.11. RegRF Bioactivity Assessment

RegRF bioactivity was assessed by its ability to reduce the count of PD-1+CD4+ T lymphocytes. Lymphocytes from the human peripheral blood of healthy volunteers were isolated in ficoll–urografin gradient, q = 1.077 g/cm^3^ (PanEco, Moscow, Russia). Cell viability determined with trypan blue was no less than 95%. The lymphocytes (2 × 10^5^ cells) were incubated with 400 µL of purified regRF (3 mg/mL, titer 1:8–1:16) in complete culture medium (RPMI-1640 supplemented with 2 mM L-glutamine, 50 U/mL penicillin and 0.05 mg/mL streptomycin), for 12 h at 37 °C in a humidified atmosphere containing 5% CO_2_. Control samples consisted of lymphocytes incubated under identical conditions, with complete culture medium added instead of regRF. The mean fluorescence intensity (MFI) of PD-1 on the surface of CD4+ T lymphocytes and the PD-1+CD4+ T lymphocyte count were calculated. For this purpose, cells were stained with an antibody cocktail consisting of Alexa Fluor 488-conjugated mouse anti-human CD3 antibodies (Abcam, Cambridge, MA, USA), PE/Cyanine7-conjugated mouse anti-CD4 antibodies and APC-conjugated mouse anti-human PD-1 antibodies (Abcam, USA) for 30 min at RT. The lymphocytes were then washed out. After that, the expression of PD-1 on CD4+ T lymphocytes was measured using laser-based flow cytometry. The strategy for measuring the specific PD-1 signal used in this study was described by us in detail previously [4].

### 2.12. Statistical Analysis

Statistical analysis was performed using GraphPad Prism 11 Software. The Shapiro–Wilk test for testing the normality of data was used. Statistical differences between groups were evaluated by a paired *t* test. Differences were considered statistically significant at *p* < 0.05.

## 3. Results

### 3.1. RegRF in the IgG Fraction of Human Blood Plasma Obtained by Ammonium Sulfate Precipitation

Given that regRF belongs to the immunoglobulin G, ammonium sulfate (AS) precipitation of blood plasma IgG was chosen as the initial step in regRF isolation. IgG was precipitated from blood plasma with AS at a concentration of 35% saturation for 1 h. Then AS was removed by ultrafiltration. RegRF detection has shown that regRF is present in the IgG fraction precipitated with 35% saturated ammonium sulfate.

### 3.2. RegRF Extraction from the IgG Fraction of Blood Plasma

The IgG fraction precipitated from blood plasma with 35% saturated ammonium sulfate contains a mixture of immunoglobulins and contaminating non-immunoglobulin proteins. We hypothesized that dissolving the IgG precipitate in ammonium sulfate at a concentration below 35% of saturation would remove part of the non-target IgG, yielding a regRF-enriched IgG fraction.

We applied two schemes for the extraction of regRF from the IgG precipitate: parallel and sequential.

In the parallel scheme, the IgG precipitate obtained from 30 mL of blood plasma at an ammonium sulfate (AS) concentration of 35% saturation was divided into three equal portions and resuspended in ammonium sulfate solutions at different concentrations: 31.5%, 29.7%, and 28% saturation. After centrifugation, the supernatants and precipitates were analyzed.

At AS concentrations of 31.5% and 29.7% of saturation, regRF was detected in both the supernatant and precipitate. RegRF was completely recovered from the precipitate at an AS concentration of 28% of saturation (Table 1). From 10 mL of blood plasma, 30 mg of regRF-enriched IgG fraction was obtained.

**Figure 1:**
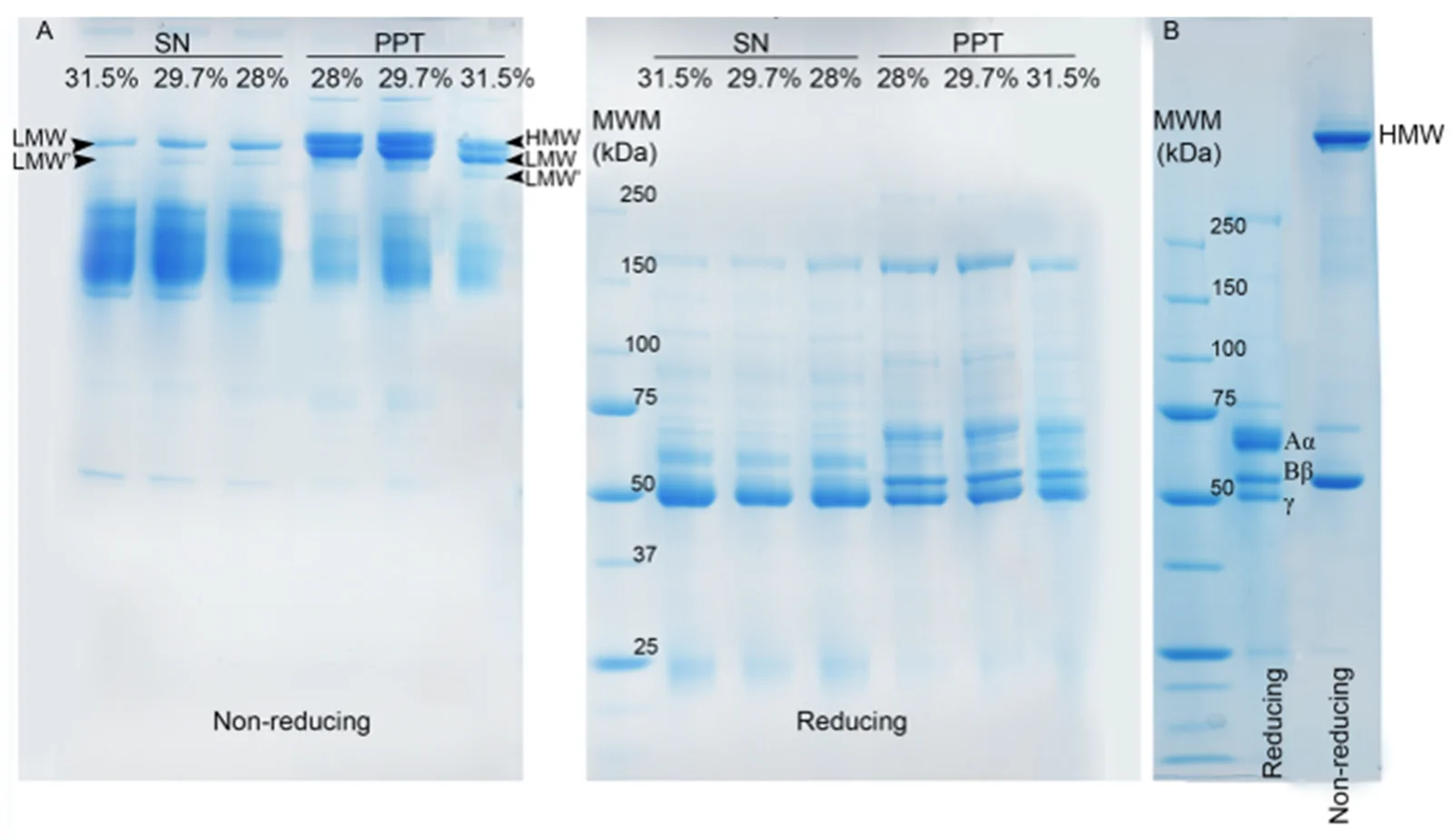
PAGE of supernatants and precipitates obtained by extraction of IgG fraction of blood plasma using a series of ammonium sulfate solutions (**A**) and fibrinogen precipitated from blood plasma with 22% saturated ammonium sulfate (**B**). PPT—precipitate. SN—supernatant. MWM—molecular weight markers. HMW—high-molecular-weight fibrinogen, LMW—low-molecular-weight fibrinogen, LMW’—LMW’ fibrinogen. SDS-PAG 3–15%.

Electrophoretic analysis of all supernatants and precipitates showed that regRF extraction with 28% saturated ammonium sulfate allowed the acquisition of regRF-enriched fraction purified from a portion of the non-target IgG and high-molecular-weight fibrinogen (HMW fibrinogen) (Figure 1A). Products of the natural partial degradation of HMW fibrinogen, namely low-molecular-weight fibrinogen (LMW) and LMW′ fibrinogen, which differ from HMW fibrinogen by the absence of a 35 kDa C-terminal polypeptide from one or two Aα chains, respectively [9,10], persisted as an undesirable contaminant in the regRF-enriched IgG fraction (Figure 1A).

To remove fibrinogen, blood plasma was subjected to preliminary precipitation with 22% saturated ammonium sulfate (AS) according to the procedure described by Colombo et al. [11]. However, only HMW fibrinogen was precipitated with 22% saturated ammonium sulfate (Figure 1B). Given that HMW fibrinogen is removed in the precipitate during regRF extraction with 28% ammonium sulfate, the preliminary 22% saturated ammonium sulfate precipitation step does not contribute to regRF purification.

The same AS concentration values—31.5%, 29.7%, and 28%—were tested in serial extraction of proteins from the IgG fraction precipitated with AS at 35% of saturation (Table 2). The serial extraction consisted of the sequential addition of AS solutions with concentrations decreasing from 31.5% to 28% of saturation to IgG precipitated with AS at 35% of saturation. Each supernatant and the final precipitate were tested for the presence of regRF.

The results of the serial extraction were consistent with those of regRF extraction from the initial IgG precipitate into ammonium sulfate solutions of varying concentrations in parallel samples. RegRF begins to partition into the solution at an ammonium sulfate concentration of 31.5% saturation. RegRF continues to dissolve at an ammonium sulfate concentration of 29.7% saturation. At 28% saturation, the remaining regRF is recovered from the precipitate (Table 2).

Therefore, precipitation of blood plasma with ammonium sulfate at 35% saturation, followed by dissolution of the precipitate in 28% saturated ammonium sulfate, allows to obtain an IgG fraction enriched in regRF.

### 3.3. Affinity of RegRF for Cibacron Blue

RegRF affinity for Cibacron Blue was discovered accidentally while fractionating human blood plasma on a BlueBeads 6FF column. RegRF was detected in the fraction eluted with a high ionic strength buffer (0.02 M potassium phosphate buffer containing 1M KCl), indicating its strong binding to Cibacron Blue. The affinity of regRF for Cibacron Blue has been demonstrated for the first time.

### 3.4. Chromatographic Purification of RegRF on Immobilized Cibacron Blue

The observed affinity of regRF for Cibacron Blue was used to purify regRF from the IgG fraction enriched in regRF.

**Figure 2:**
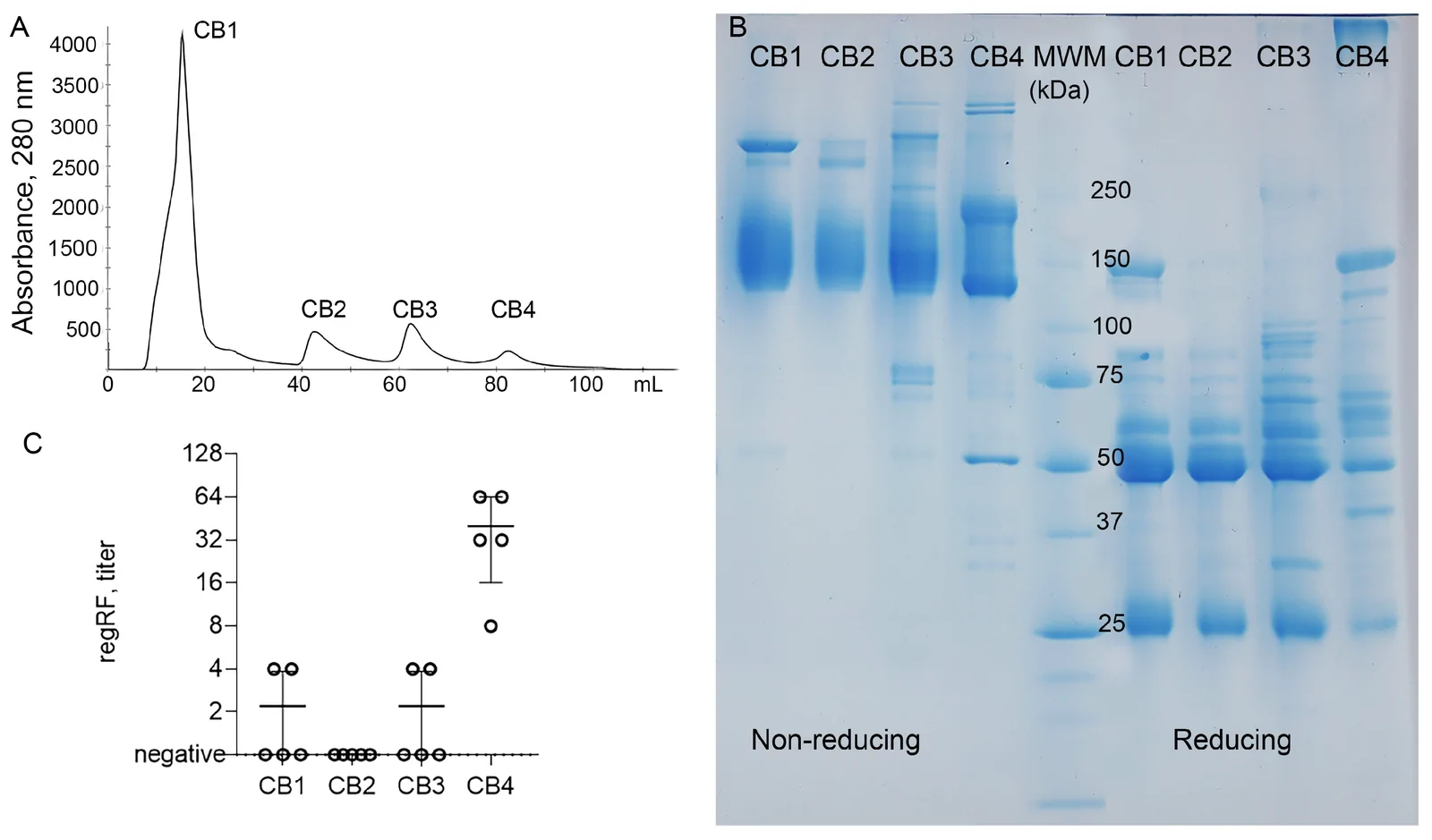
Purification of regRF-enriched IgG fraction using BlueBeads 6FF column. (**A**). Elution profile. Column was equilibrated with 0.02 M potassium phosphate buffer, pH 7.4. Fractions were eluted stepwise with 5%, 20%, 50% and 100% of 2 M KCl. Flow rate: 1.5 mL/min. (**B**). PAGE of fractions eluted from BlueBeads 6FF column. (**C**). Detection of regRF in fractions. Results are shown for five independently prepared samples. CB1—flow-through IgG fraction, CB2—IgG fraction eluted with 0.1 M KCl, CB3—IgG fraction eluted with 0.4 M KCl, CB4—IgG fraction eluted with 1M KCl.

The elution profile of the regRF-enriched IgG fraction from the BlueBeads 6FF column, together with the electrophoretic analysis of the eluted fractions (CB1–CB4), is shown in Figure 2A and Figure 2B, respectively.

As expected, regRF was detected in the CB4 fraction with a high affinity for Cibacron Blue (Figure 2C). The CB4 fraction contained ~3% of the loaded protein. The CB1 (flow-through) and CB3 (eluted with 0.4 M KCl) fractions contained small amounts of regRF in some samples (Figure 2C).

In addition to the low-ionic-strength potassium phosphate buffer, 0.15 M PBS (pH 7.4) was tested as a binding buffer, which did not alter the binding and purification efficiency of regRF. For elution, both a linear gradient and several step elution variants using KCl were evaluated. The elution flow rate was also varied from 1.5 to 5 mL/min. The highest number of resolved fractions and the maximum yield of regRF were observed with stepwise elution using 5%, 20%, 50%, and 100% of 2 M KCl at a flow rate of 1.5 mL/min.

Electrophoretic analysis showed that chromatography on immobilized Cibacron Blue allows the purification of regRF from non-target IgG, which is distributed across the first three fractions (Figure 2B). In addition, chromatography on immobilized Cibacron Blue allowed purification of regRF from LMW and LMW’ fibrinogen, which remains in the CB1-CB3 fractions (Figure 2B).

### 3.5. RegRF Has No Affinity for Protein A

PAGE under reducing conditions revealed that the regRF-containing CB4 fraction differs from the other three regRF-free fractions by the presence of a protein with Mr ~ 60–65 kDa (Figure 2B), which may correspond to the heavy chain of IgG3. This led us to assume that regRF might belong to the IgG3 subclass. In this case, regRF can be separated from non-target IgG subclasses using chromatography on Protein A that exhibits no affinity to human IgG3 but binds to other human IgG subclasses over the pH range of 6.0–7.0.

The regRF affinity for Protein A was evaluated. The regRF-enriched IgG fraction was applied in the binding buffer—0.15 M PBS, pH 7.0.

**Figure 3:**
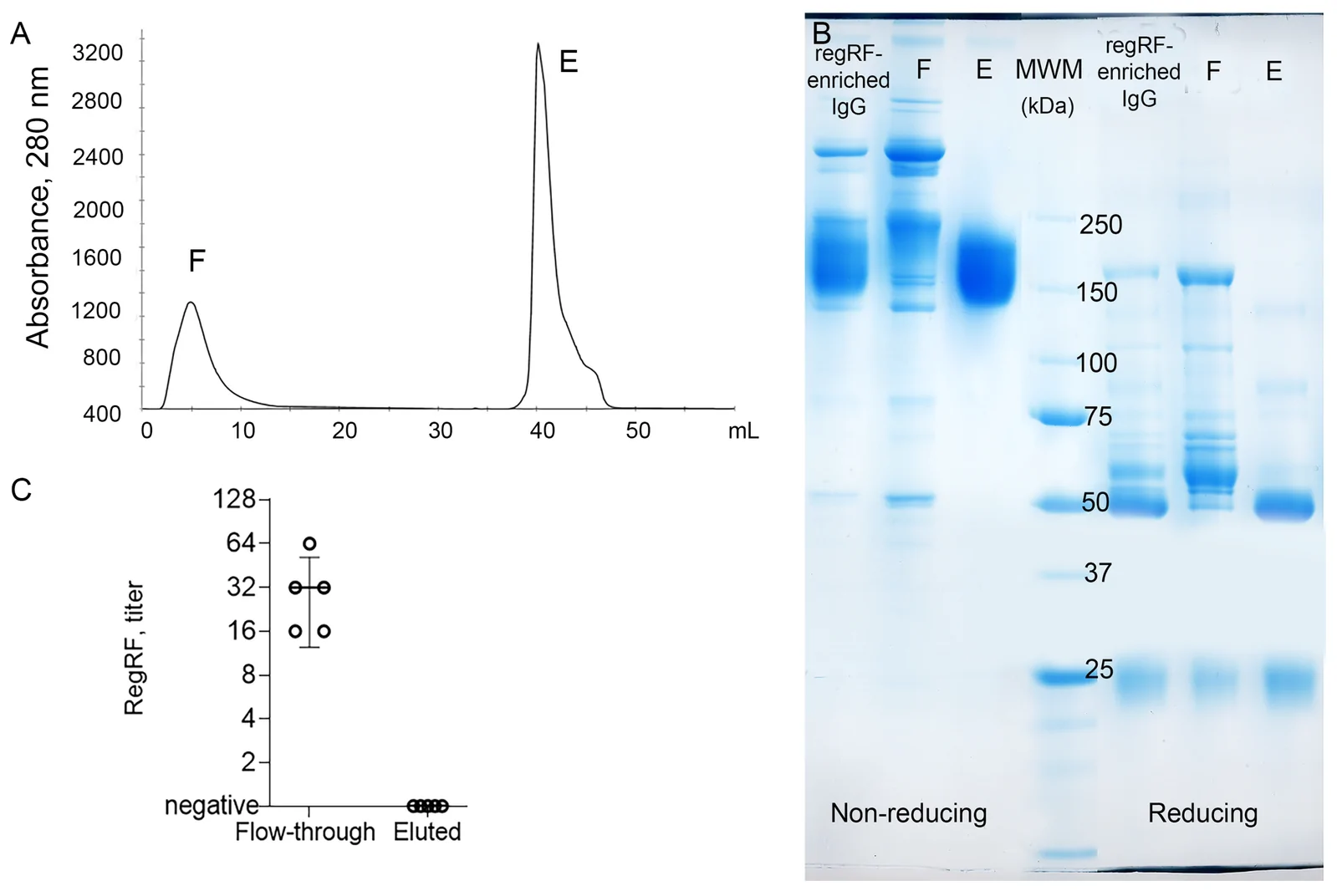
Purification of the regRF-enriched IgG fraction using a Protein A Sepharose column. F—flow-through fraction. E—eluted fraction. (**A**). Elution profile. Binding buffer: 0.15 M PBS, pH 7.0, elution buffer: 0.1 M glycine-HCl buffer, pH 2.7. Flow rate: 5 mL/min. (**B**). PAGE of the flow-through and eluted fractions. (**C**). Detection of regRF in fractions. Results are shown for five independently prepared samples.

RegRF was detected in the flow-through fraction, showing no binding to Protein A (Figure 3). The flow-through fraction contained ~35% of the loaded protein. Electrophoretic analysis of the flow-through and eluted fractions showed that Protein A chromatography allowed the purification of regRF from most non-target IgG. However, the regRF-containing fraction was contaminated with LMW-fibrinogen and LMW’-fibrinogen (Figure 3B).

### 3.6. Purification of RegRF by Sequential Cibacron Blue and Protein A Chromatography

**Figure 4:**
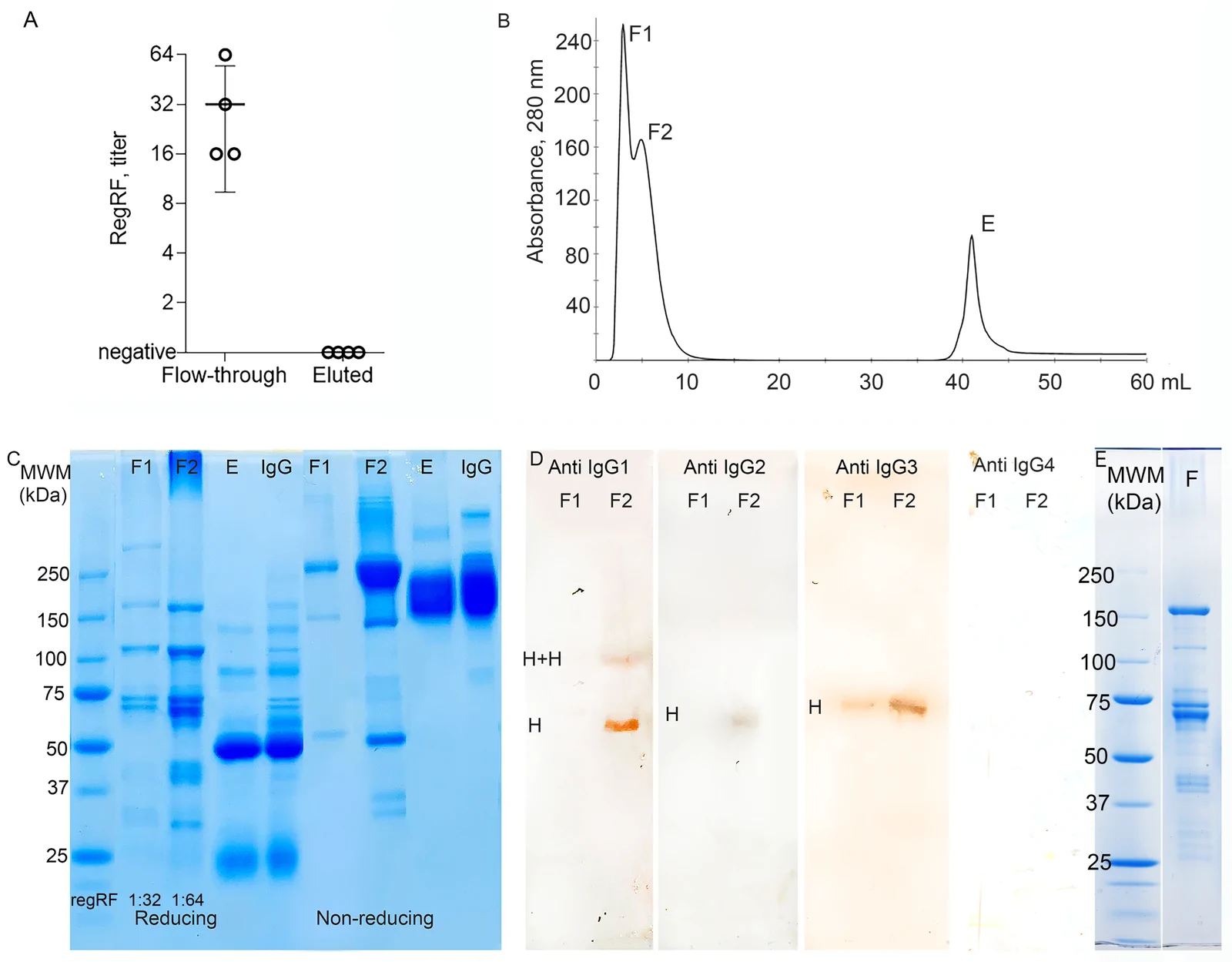
Fractionation of the regRF-containing CB4 fraction on a Protein A Sepharose column. (**A**). Detection of regRF in the fractions obtained from the Protein A Sepharose column. The protein concentration in the samples was 2.5–4 mg/mL. (**B**). Elution profile of the CB4 fraction loaded onto the Protein A Sepharose column at a total protein amount of 0.8 mg. The binding buffer: 0.15 M PBS, KCl (κ = 86 mSm/cm), pH 7.0. The elution buffer: 0.1 M glycine-HCl buffer, pH 2.7. Flow rate: 5 mL/min. (**C**). PAGE of fractions obtained during separation of the CB4 fraction loaded onto a Protein A Sepharose column at a total protein amount of 0.8 mg. (**D**). Identification of IgG isotypes in regRF-containing flow through fractions by Western blotting. (**E**). PAGE of the unsplit RegRF-containing flow-through peak. F, F1 and F2—flow-through fractions, E—eluted fraction. SDS-PAG 3–15%. H—heavy chains.

RegRF samples obtained from Cibacron Blue (Figure 2B) and Protein A (Figure 3B) sorbents exhibited varying impurity profiles. Therefore, these chromatographic runs were carried out sequentially. A RegRF-enriched fraction was applied to the Cibacron Blue column. The eluted CB4 fraction containing regRF was then fractionated using a Protein A Sepharose column. RegRF was detected in the flow-through peak (Figure 4A).

When a small amount of the CB4 fraction (containing 0.8 mg of protein) was applied to the Protein A Sepharose column, the flow-through peak split into two unresolved peaks, F1 and F2 (Figure 4B). RegRF was detected in both flow-through peaks (Figure 4C).

Western blotting showed that bands with molecular masses ~600 kDa under reducing conditions in the regRF-containing flow-through fractions F1 and F2 corresponded to IgG (Figure 4D). However, the IgG subclass composition differed between the F1 and F2 fractions. The F1 fraction showed a band corresponding only to IgG3 (Figure 4D), whereas the F2 fraction revealed bands corresponding to IgG3, non-classical heavy chains of IgG1, as well as IgG1 heavy-chain dimers and trace amounts of non-classical heavy chains of IgG2 (Figure 4D). The non-classical heavy chains of IgG1 and IgG2 exhibited lower electrophoretic mobility than their classical counterparts (Figure 4D). The presence of IgG1 and IgG2, in addition to IgG3, in the regRF-containing F2 fraction does not exclude the possibility that regRF may be represented not only by IgG3 but also by IgG1 and IgG2. Therefore, the subclass profile of regRF remains to be characterized.

Densitometric analysis of the unsplit regRF-containing flow-through fraction (Figure 4E) revealed that it contained 59% IgG. RegRF remained contaminated with α2-macroglobulin (a band with an Mr of 180 kDa under reducing conditions), a protein migrating as a 70 kDa band and proteins forming bands with Mr values of 40–45 kDa under reducing conditions, which accounted for 18.5%, 10.5% and 12%, respectively (Figure 4E).

Thus, chromatography on immobilized Cibacron Blue followed by purification of the regRF-containing fraction using Protein A Sepharose enables the isolation of relatively pure regRF. The regRF purification method requires further optimization to remove α2-macroglobulin.

### 3.7. Specificity to PD-1 and Bioactivity of Purified RegRF

RegRF, purified by sequential Cibacron Blue and Protein A chromatography, was tested for PD-1 specificity and bioactivity.

**Figure 5:**
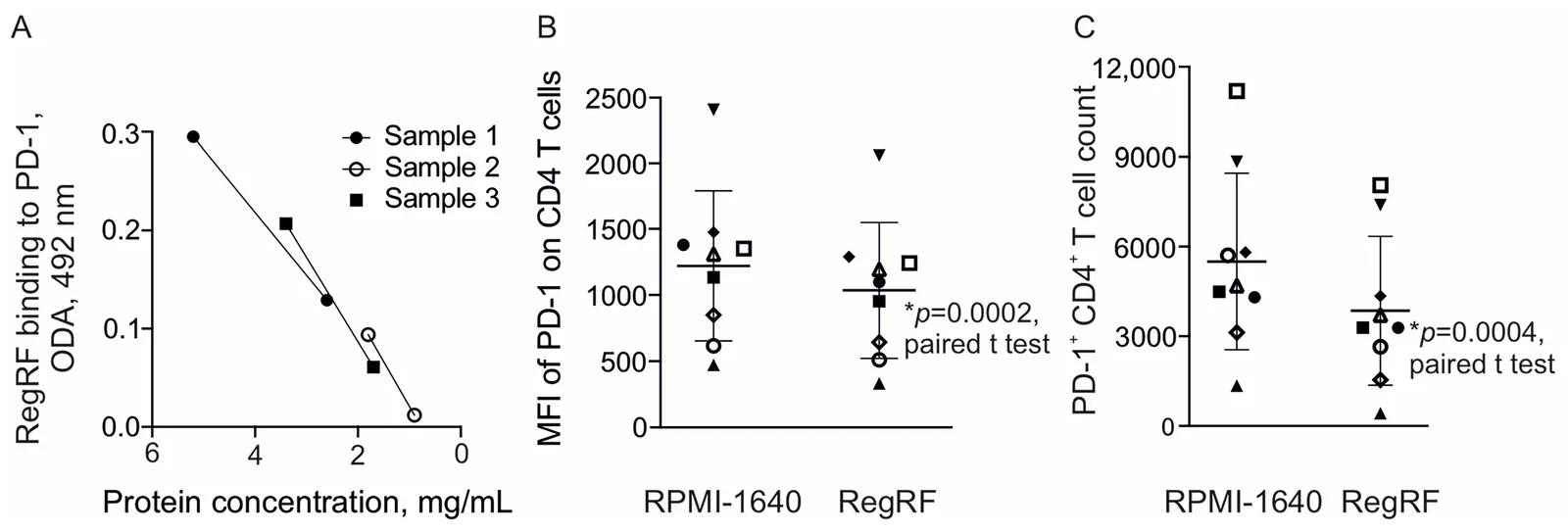
Assessment of the specificity and bioactivity of purified regRF. (**A**). Binding of regRF to PD-1. Three independently obtained purified regRF samples were tested. (**B**). Effect of regRF on the MFI of PD-1 on CD4+ T lymphocytes. (**C**). Effect of regRF on the PD-1-expressing CD4+ T lymphocyte count. The purity of the regRF samples was approximately 60%. Lymphocytes were obtained from 9 healthy volunteers. Lymphocytes from the same individual are indicated by the same marker shape. Each of three independently obtained purified regRF samples was tested on lymphocytes from three individuals (* *p* < 0.05).

RegRF bound to PD-1, indicating conserved regRF specificity for the PD-1 molecule (Figure 5A).

RegRF is known to exert a cytotoxic effect on activated CD4+ T lymphocytes through the PD-1 pathway [4]. Therefore, regRF bioactivity was evaluated by its effect on PD-1-expressing CD4+ T lymphocytes. The treatment of lymphocytes with the purified regRF was shown to decrease the mean fluorescence intensity (MFI) of PD-1 on CD4+ T lymphocytes (Figure 5B) and PD-1+CD4+ T lymphocyte count (Figure 5C). The regRF-depleted IgG-containing CB4 fraction was used as a control for the non-specific action of IgG on PD-1+CD4+ T lymphocytes. Its effect on the cells did not differ from that of the RPMI-1640 medium.

Therefore, sequential chromatography on Cibacron Blue and Protein A allows the isolation of bioactive regRF.

## 4. Discussion

The current study aimed to select methods, sorbents, and conditions suitable for the isolation of bioactive regulatory rheumatoid factor from blood plasma.

For crude purification, we tested IgG precipitation from plasma with ammonium sulfate and subsequent extraction of regRF. Ammonium sulfate’s ability to preserve native protein conformation was taken into account [12]. Extraction conditions were optimized to maximize regRF recovery and remove a portion of non-target IgG and high-molecular-weight fibrinogen.

RegRF showed a lack of affinity for Protein A. The discovered lack of regRF binding to protein A allows the purification of regRF from non-target immunoglobulins without the use of harsh elution conditions associated with denaturation, alteration of antigen-binding behavior, and conformational changes leading to aggregation [13]. We demonstrated that regRF isolation on Protein A Sepharose can be performed under physiological conditions in 0.15 M PBS, pH 7.0.

It is well known that all human IgG subclasses, except IgG3, bind to Protein A [14]. The lack of regRF affinity for Protein A led us to assume that regRF belongs to IgG3. However, in addition to IgG3, IgG1 and IgG2 with unusually low electrophoretic mobility on PAG were detected in the purified regRF sample (Figure 4). Therefore, it cannot be ruled out that the regRF subclass profile is also represented by IgG1 and IgG2 lacking Protein A affinity. Thus, the elucidation of the regRF subclass profile requires further studies.

During the development of the regRF purification method, regRF’s affinity for Cibacron Blue was discovered. Binding to Cibacron Blue has previously been demonstrated for many proteins and enzymes which could not be purified or fractionated by other means [15]. The anthraquinone and the terminal phenylsulfonate rings of Cibacron Blue can be oriented like the adenine and nicotinamide rings of NAD, respectively. The two internal rings of the dye mimic the two ribofuranose rings of NAD. Two of the dye’s anionic sulfonate groups are positioned similarly to the two anionic phosphates of NAD [15]. Therefore, enzymes possessing the Rossmann fold (NAD-binding domain) undergo structure-specific affinity interactions with Cibacron Blue [15,16]. Cibacron Blue can also interact with proteins through nonspecific electrostatic and hydrophobic interactions [15,17,18,19]. The binding of regRF to Cibacron Blue under low ionic strength conditions and the efficient elution of regRF with a high concentration of KCl suggest a contribution of electrostatic interactions between regRF and Cibacron Blue.

Chromatography on Cibacron Blue allows the purification of regRF not only from non-target IgG but also from low-molecular-weight fibrinogen.

The combination of Cibacron Blue and Protein A chromatography yields sufficiently pure regRF.

Assessment of the specificity of purified regRF for PD-1 and its cytotoxic effect on PD-1–expressing CD4+ T lymphocytes demonstrated that the applied purification methods preserve regRF specificity and biological activity.

## 5. Conclusions

We identified previously unknown physicochemical properties of regRF that can be used for its purification from human blood plasma. In particular, regRF exhibited affinity for Cibacron Blue and lacked Protein A binding. The combination of Cibacron Blue and Protein A chromatography yields sufficiently pure regRF. Purified regRF exhibited binding to PD-1 and a cytotoxic effect on PD-1-expressing CD4+ T lymphocytes. Therefore, the developed method for regRF isolation enables the production of biologically active regRF.

## Figures and Tables

**Table 1:** RegRF in IgG fractions obtained by dissolving the sulfate precipitate of blood plasma in 31.5%, 29.7%, and 28% saturated ammonium sulfate.

	Supernatant			Precipitate		
Ammonium Sulfate Concentration for regRF Extraction, % of Saturation	Total Protein Concentration (mg/mL)	Total Protein Yield (mg) from 10 mL of Plasma	RegRF, Titer	Total Protein Concentration, mg/mL	Total Protein Yield (mg) from 10 mL of Plasma	RegRF, Titer
31.5	8.2	16.4	1:16	17.7	35.5	1:16
29.7	11.8	23.6	1:32	12.1	24.2	1:16
28.0	15.1	30.2	1:64	11.0	22.0	Not detected

**Table 2:** RegRF in IgG fractions obtained from the sulfate precipitate of blood plasma by serial extraction with ammonium sulfate solutions of decreasing concentration.

	Ammonium Sulfate Concentration for regRF Extraction, % of Saturation	Total Protein Concentration, mg/mL	Total Protein Yield (mg) from 10 mL of Plasma	RegRF, Titer
Supernatants	31.5	8.4	16.8	1:32
	29.7	5.4	10.8	1:8
	28.0	2.4	4.8	1:2
Final precipitate		7.6	15.2	1:2

## Data Availability

The data that support the findings of this study are available from the corresponding author upon reasonable request.

## Supplementary Materials

The following supporting information can be downloaded at: https://www.mdpi.com/article/10.3390/antib15050098/s1. Figure S1: Scheme of regRF extraction from IgG fraction obtained by precipitation of blood plasma with 35% saturated ammonium sulfate. A. Parallel extraction. B. Sequential extraction.

## Abbreviations

The following abbreviations are used in this manuscript:

AS	Ammonium sulfate
CB1	Flow-through IgG fraction from BlueBeads 6FF column
CB2	IgG fraction eluted with 0.1 M KCl from BlueBeads 6FF column
CB3	IgG fraction eluted with 0.4 M KCl from BlueBeads 6FF column
CB4	IgG fraction with affinity for Cibacron Blue
HMW	High-molecular-weight fibrinogen
LMW	Low-molecular-weight fibrinogen
MFI	Mean fluorescence intensity
MWM	Molecular weight markers
PPT	Precipitate
RegRF	Regulatory rheumatoid factor
SN	Supernatant
