# Supplementary material for: Purification of Bioactive Natural Antibodies to PD-1 Known as Regulatory Rheumatoid Factor from Human Blood Plasma
Source: Antibodies (Basel). 2026 Oct 19;15(5):98. doi: 10.3390/antib15050098 (PMC13700785; doi:10.3390/antib15050098)
Supplement: Supplementary file 1 [file antibodies-15-00098-s001.zip › antibodies-4540523-supplementary.pdf]

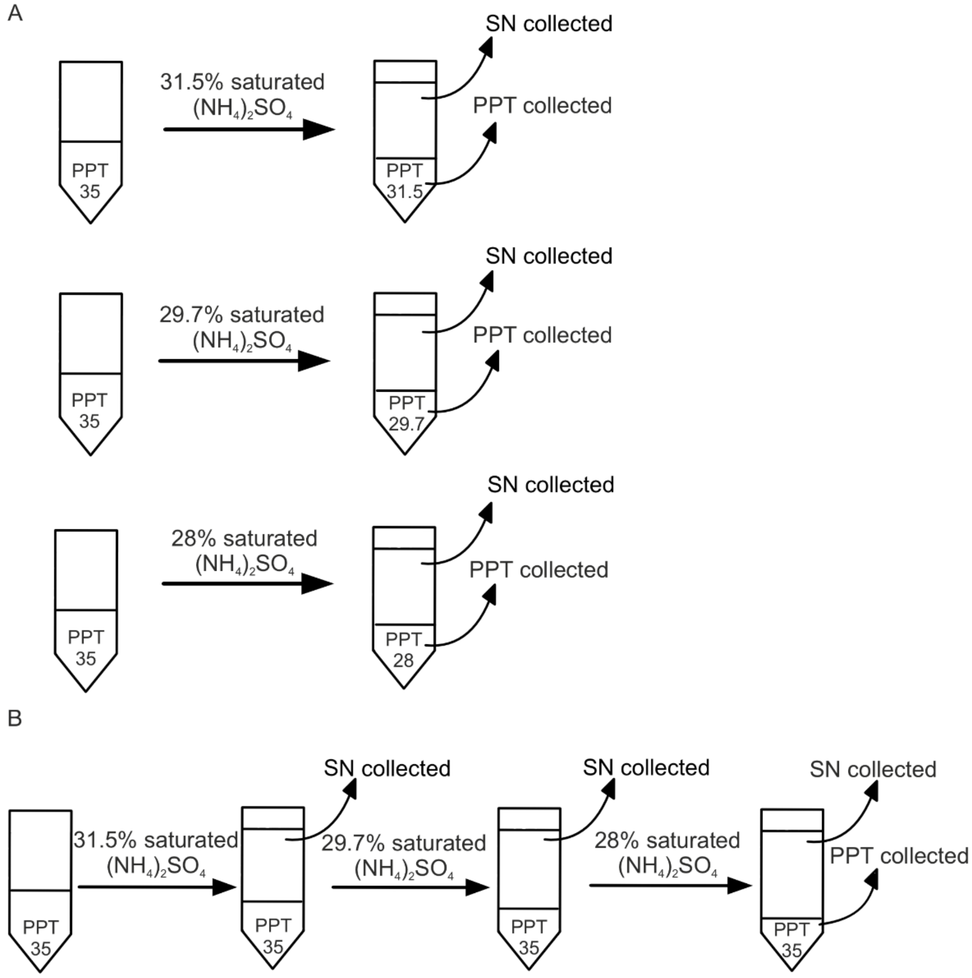

Figure S1. Scheme of regRF extraction from IgG fraction obtained by precipitation of blood plasma with 35% saturated ammonium sulfate. A. Parallel extraction. B. Sequential extraction.
